# Correction: Trace element concentrations in forage seagrass species of *Chelonia mydas* along the Great Barrier Reef

**DOI:** 10.1371/journal.pone.0292777

**Published:** 2023-10-05

**Authors:** Adam Wilkinson, Ellen Ariel, Jason van de Merwe, Jon Brodie

There are errors in the Funding section. The correct Funding statement is: Field and laboratory analysis components were kindly funded by the Worldwide Fund for nature (WWF) Australia. Publication costs were funded through the Higher Degree by Research Enhancement scheme (HDRES) offered by the College of Public Health, Medicine and Veterinary Sciences, James Cook University.
