# Supplementary material for: Trace element concentrations in forage seagrass species of Chelonia mydas along the Great Barrier Reef
Source: PLoS One. 2022 Jun 15;17(6):e0269806. doi: 10.1371/journal.pone.0269806 (PMC9200345; doi:10.1371/journal.pone.0269806)
Supplement: S1 Fig — (DOCX) [file pone.0269806.s001.docx]

**S1 Fig 1. RAW data analysed and reported in research article “Trace element concentrations in forage seagrass species of *Chelonia mydas* along the Great Barrier Reef”**

| Site -sample # | Al | Cd | Co | Cu | Fe | Mg | Mn | Ni | Pb | Zn | location | event |
| --- | --- | --- | --- | --- | --- | --- | --- | --- | --- | --- | --- | --- |
| CB-01 | 2996.14 | 0.38 | 1.31 | 4.15 | 2102.41 | 7403.92 | 411.84 | 3.04 | 1.12 | 21.67 | CB | Before |
| CB-02 | 1766.75 | 0.24 | 0.9 | 4.35 | 1683.57 | 7238.45 | 328.87 | 3.43 | 0.25 | 19.89 | CB | Before |
| CB-03 | 1642.27 | 0.3 | 1.5 | 3.36 | 1381.93 | 6830.9 | 358.3 | 2.3 | 0.25 | 18.14 | CB | Before |
| CB-04 | 3006.93 | 0.32 | 1.48 | 5.25 | 2897.78 | 7511.67 | 333.15 | 3.71 | 1.09 | 28.86 | CB | Before |
| CB-05 | 4469.56 | 0.24 | 1.89 | 5.37 | 3349.59 | 7499.7 | 464.63 | 5.01 | 1.72 | 27.42 | CB | Before |
| CB-06 | 3399.21 | 0.2 | 1.31 | 3.7 | 2658.56 | 7040.82 | 481.37 | 3.17 | 0.25 | 23 | CB | Before |
| CB-07 | 3746.68 | 0.36 | 1.51 | 3.81 | 2563.51 | 7376.53 | 422.57 | 3.16 | 1.75 | 19.29 | CB | Before |
| CB-08 | 6786.71 | 0.45 | 2.19 | 5.76 | 4540.93 | 7308.35 | 395.84 | 4.95 | 1.79 | 22.9 | CB | Before |
| CB-09 | 12920.98 | 0.77 | 3.09 | 6.4 | 8392.73 | 7184.22 | 392.65 | 7.76 | 4.36 | 31.27 | CB | Before |
| CB-10 | 3962.47 | 0.34 | 1.46 | 4.16 | 2856.92 | 7012.9 | 354.83 | 3.21 | 1.26 | 17.38 | CB | Before |
| CB-12 | 3124.18 | 0.43 | 1.68 | 9.96 | 4214.76 | 8240.29 | 238.98 | 2.75 | 0.99 | 56.03 | CB | Before |
| CB-13 | 2264.53 | 0.33 | 1.36 | 5.93 | 3230.66 | 8675.79 | 248.49 | 2.71 | 1.86 | 35.47 | CB | Before |
| CB-14 | 3298 | 0.39 | 1.85 | 1.85 | 4046.66 | 8251.65 | 286.99 | 2.98 | 1.22 | 42.58 | CB | Before |
| CB-15 | 3964.55 | 0.41 | 1.93 | 6.67 | 3916.8 | 8686.55 | 338.3 | 3.22 | 2.49 | 39.36 | CB | Before |
| CB-16 | 3576.44 | 0.38 | 2.36 | 7.03 | 3916.69 | 8409.32 | 411.34 | 3.98 | 2.06 | 30.42 | CB | Before |
| CB-17 | 2629.39 | 0.35 | 1.89 | 9.61 | 3295.35 | 8688.74 | 321.1 | 3.39 | 2.87 | 33.21 | CB | Before |
| CB-18 | 1988.56 | 0.34 | 2.11 | 8.61 | 2437.77 | 8557.97 | 247.36 | 3.41 | 2.27 | 30.65 | CB | Before |
| CB-A1 | 2377.48 | 0.45 | 1.02 | 5.81 | 1782.49 | 7735.96 | 305.79 | 2.43 | 0.25 | 19.34 | CB | After |
| CB-A2 | 651.36 | 0.3 | 0.19 | 3.65 | 556.88 | 7008.57 | 149.7 | 1.71 | 0.25 | 16.09 | CB | After |
| CB-A4 | 4018 | 0.39 | 1.05 | 3.97 | 4603.01 | 7459.46 | 121.57 | 4.04 | 2.17 | 24.89 | CB | After |
| CB-A5 | 2660.69 | 0.47 | 1 | 4.48 | 2530.46 | 7127.79 | 263.4 | 3.8 | 1.22 | 23.61 | CB | After |
| CB-A6 | 2433.19 | 0.34 | 0.85 | 3.94 | 1758.59 | 6998.19 | 229.15 | 2.8 | 0.25 | 18.53 | CB | After |
| CB-A7 | 4975.87 | 0.44 | 1.76 | 4.81 | 3516.77 | 6835.17 | 250.9 | 4.2 | 1.78 | 25.32 | CB | After |
| CB-A8 | 6544.2 | 0.53 | 1.8 | 5.22 | 4602.96 | 7209.99 | 216.88 | 6.06 | 2.11 | 28.35 | CB | After |
| CB-A9 | 5728.26 | 0.52 | 2.1 | 4.1 | 4611.08 | 6544.14 | 288.1 | 4.81 | 1.8 | 24.88 | CB | After |
| CB-A10 | 3085.25 | 0.4 | 1.01 | 4.12 | 2742.83 | 7056.37 | 144.81 | 3.97 | 0.25 | 24.82 | CB | After |
| CB-A11 | 2845.35 | 0.34 | 0.74 | 2.79 | 2852.53 | 7176.86 | 157.45 | 2.98 | 0.69 | 22.14 | CB | After |
| CB-A12 | 7083.3 | 0.6 | 1.93 | 4.7 | 5207.82 | 7238.27 | 289.04 | 5.46 | 2.14 | 25.52 | CB | After |
| CB-A13 | 5728.26 | 0.52 | 2.1 | 4.1 | 4263.6 | 7494.4 | 325.46 | 4.01 | 1.57 | 30.29 | CB | After |
| CB-A14 | 2442.6 | 0.36 | 1.16 | 3.17 | 2090.84 | 6916.33 | 289.34 | 2.7 | 0.7 | 27.94 | CB | After |
| UB-01 | 1542.61 | 0.29 | 1.86 | 5.52 | 1313.8 | 8236.38 | 206.23 | 5.78 | 0.25 | 14.41 | UB | Before |
| UB-02 | 1359.83 | 0.25 | 1.78 | 4.94 | 1334.89 | 9258.18 | 194.46 | 3.31 | 0.25 | 10.41 | UB | Before |
| UB-03 | 1491.95 | 0.25 | 1.57 | 5.17 | 1520.46 | 8652.37 | 166.1 | 3.04 | 0.93 | 12.24 | UB | Before |
| UB-04 | 1636.38 | 0.24 | 2.15 | 6.26 | 1376.33 | 8573.64 | 196.33 | 3.68 | 0.25 | 13.14 | UB | Before |
| UB-05 | 818.64 | 0.23 | 1.4 | 4.85 | 930.84 | 9001.13 | 145.73 | 3.07 | 0.25 | 11.95 | UB | Before |
| UB-06 | 3217.57 | 0.29 | 2.54 | 4.93 | 2595.87 | 8215.02 | 179.64 | 6.63 | 0.93 | 14.8 | UB | Before |
| UB-07 | 3967.05 | 0.38 | 2.71 | 5.33 | 2980.22 | 8898.21 | 283.22 | 5.09 | 1.57 | 11.5 | UB | Before |
| UB-08 | 1965.98 | 0.22 | 2.01 | 5.03 | 2082.28 | 8380.72 | 143.77 | 5.9 | 0.25 | 12.81 | UB | Before |
| UB-09 | 1056.52 | 0.17 | 1.35 | 5.23 | 1090.83 | 8769.12 | 192.49 | 2.71 | 0.25 | 18.02 | UB | Before |
| UB-10 | 915.69 | 0.21 | 1.1 | 4.35 | 1017.82 | 8112.65 | 276.32 | 2.33 | 0.25 | 13.13 | UB | Before |
| UB-11 | 2249.65 | 0.3 | 2.12 | 5.35 | 2215.83 | 8484.9 | 593.19 | 4.72 | 0.25 | 16.92 | UB | Before |
| UB-12 | 1323.38 | 0.24 | 1.84 | 4.23 | 1846.44 | 10108.94 | 256.14 | 3.56 | 0.25 | 11.73 | UB | Before |
| UB-13 | 688.65 | 0.16 | 1.5 | 4.4 | 1196.35 | 8974.6 | 374.48 | 3.56 | 0.25 | 12.1 | UB | Before |
| UB-14 | 1891.19 | 0.29 | 2.25 | 5.18 | 2377.73 | 8919.31 | 257.3 | 7.21 | 0.25 | 14.34 | UB | Before |
| UB-15 | 1768.4 | 0.56 | 1.81 | 5.03 | 5435.76 | 6671.73 | 221.11 | 3.14 | 1.07 | 30.34 | UB | Before |
| UB-A1 | 1276.01 | 0.37 | 1.27 | 5.32 | 2683.92 | 7236.42 | 153.08 | 2.92 | 0.25 | 23.88 | UB | After |
| UB-A2 | 1233.48 | 0.35 | 1.56 | 6.61 | 1754.86 | 7725.36 | 417.8 | 3.67 | 0.25 | 26.54 | UB | After |
| UB-A3 | 1813.13 | 0.44 | 2.26 | 8.51 | 4035.36 | 9123.96 | 320.64 | 5.4 | 0.25 | 26.66 | UB | After |
| UB-A4 | 2592.42 | 0.58 | 2.64 | 6.12 | 6372.35 | 6898.12 | 208.54 | 5.66 | 1.72 | 26.77 | UB | After |
| UB-A5 | 1091.92 | 0.45 | 0.85 | 4.93 | 3743.63 | 7661.65 | 151.27 | 2.77 | 0.25 | 27.47 | UB | After |
| UB-A6 | 1792.98 | 0.42 | 2.29 | 10.32 | 2601.44 | 7017.44 | 397.08 | 4.43 | 0.67 | 35.26 | UB | After |
| UB-A7 | 2360.2 | 0.48 | 2.85 | 9.46 | 3246.58 | 7467.17 | 359.36 | 4.17 | 0.25 | 28.93 | UB | After |
| UB-A8 | 1910.52 | 0.57 | 2.27 | 7.34 | 4378.61 | 7716.38 | 344.95 | 4.48 | 0.9 | 24.68 | UB | After |
| UB-A9 | 1425.15 | 0.44 | 2.59 | 8.44 | 2288.64 | 7659.51 | 576.34 | 3.32 | 0.93 | 23.56 | UB | After |
| UB-A10 | 1571.39 | 0.51 | 2.29 | 8.56 | 2914.54 | 7180.87 | 388.08 | 3.94 | 0.94 | 29.99 | UB | After |
| UB-A11 | 1385.49 | 0.61 | 2.14 | 5.32 | 7149.3 | 6784.29 | 150.48 | 5.04 | 1.71 | 49.8 | UB | After |
| UB-A12 | 664.62 | 0.49 | 2.81 | 4.78 | 5319.57 | 6620.6 | 232.4 | 2.17 | 1.27 | 30.38 | UB | After |
| UB-A13 | 620.55 | 0.28 | 2.35 | 5.71 | 2651.1 | 6482.77 | 326.31 | 3.19 | 0.25 | 20.05 | UB | After |
| UB-A14 | 822.14 | 0.32 | 1.8 | 12.13 | 1545.89 | 8272.84 | 204.94 | 5.02 | 0.25 | 24.01 | UB | After |
| UB-A15 | 1500.35 | 0.31 | 2.64 | 4.73 | 1860.84 | 9032.78 | 339.04 | 6.95 | 1.02 | 11.39 | UB | After |
| EB-01 | 1309.72 | 0.57 | 2.66 | 2.95 | 4718.52 | 6191.88 | 450.74 | 5 | 0.25 | 14.72 | EB | Before |
| EB-03 | 1063.05 | 0.21 | 0.56 | 1.96 | 1917.47 | 6843.11 | 40.47 | 3.05 | 0.25 | 18.91 | EB | Before |
| EB-09 | 2416.88 | 0.42 | 2.25 | 2.89 | 4212.22 | 7197.75 | 290.85 | 2.79 | 0.25 | 24.26 | EB | Before |
| EB-11 | 3112.23 | 0.39 | 1.8 | 2.46 | 4592.16 | 7282.79 | 207.79 | 4.73 | 2.05 | 19.13 | EB | Before |
| EB-13 | 2694.04 | 0.35 | 1.77 | 2.6 | 3439.92 | 7893.85 | 244.07 | 7.54 | 0.25 | 23.76 | EB | Before |
| EB-16 | 2622.77 | 0.36 | 1.57 | 3.87 | 3851.2 | 8267.65 | 260.28 | 5.51 | 0.25 | 28.96 | EB | Before |
| EB-22 | 1566.8 | 0.28 | 1.41 | 3.3 | 1999.71 | 7882.88 | 218.59 | 3.72 | 0.25 | 16.08 | EB | Before |
| EB-28 | 1047.3 | 0.31 | 1.08 | 4.09 | 2993.15 | 7807.87 | 160.41 | 3.14 | 0.25 | 23.65 | EB | Before |
| EB-30 | 960.71 | 0.22 | 0.89 | 3.16 | 2073.5 | 7719.73 | 212.11 | 2.21 | 0.25 | 16.31 | EB | Before |
| EB-32 | 1731.18 | 0.24 | 0.91 | 2.72 | 2112.83 | 6947.39 | 108.08 | 1.85 | 1.11 | 24.07 | EB | Before |
| EB-34 | 1777.71 | 0.24 | 0.78 | 2.6 | 2440.06 | 7914.88 | 97.25 | 2.99 | 0.25 | 13.33 | EB | Before |
| EB-A1 | 2617.15 | 0.32 | 0.97 | 2.6 | 2912.35 | 7484.94 | 152.09 | 5.64 | 0.25 | 12.25 | EB | After |
| EB-A2 | 1800.77 | 0.29 | 0.71 | 2.53 | 3072.94 | 6740.67 | 78.18 | 3.36 | 0.66 | 10.28 | EB | After |
| EB-A3 | 2644.11 | 0.32 | 0.9 | 2.04 | 2929.8 | 6799.1 | 126.95 | 3 | 0.25 | 11.82 | EB | After |
| EB-A4 | 2161.37 | 0.43 | 1.65 | 4.22 | 4654.35 | 7444.67 | 266.84 | 5.45 | 0.25 | 19.33 | EB | After |
| EB-A5 | 2727.01 | 0.35 | 1 | 2.05 | 3932.63 | 7011.42 | 94.01 | 8.01 | 0.25 | 11.38 | EB | After |
| EB-A6 | 1901.84 | 0.33 | 0.85 | 2.33 | 2979.43 | 6064.93 | 125.72 | 4.28 | 0.25 | 11.8 | EB | After |
| EB-A7 | 2478.68 | 0.41 | 1.77 | 3.27 | 4481.17 | 7564.2 | 253.86 | 6.05 | 0.25 | 19.55 | EB | After |
| EB-A8 | 1268.88 | 0.19 | 0.56 | 1.4 | 2265.94 | 6618.66 | 42.3 | 5.07 | 0.25 | 10.66 | EB | After |
| EB-A9 | 2295.68 | 0.31 | 1.07 | 2.67 | 2720.8 | 7332.39 | 160.56 | 7.12 | 0.95 | 12.66 | EB | After |
| EB-A10 | 1885.26 | 0.28 | 0.96 | 2.86 | 2146.75 | 7394.8 | 60.83 | 6.7 | 0.25 | 15.11 | EB | After |
| EB-A11 | 1438.39 | 0.19 | 0.94 | 2.19 | 2000.76 | 6479.89 | 33.9 | 5.76 | 0.25 | 20.56 | EB | After |
| EB-A12 | 869.42 | 0.11 | 0.38 | 2.07 | 1034.35 | 8021.17 | 65.85 | 3.54 | 0.76 | 18.3 | EB | After |
| EB-A13 | 1294.33 | 0.18 | 0.8 | 2.12 | 2094.98 | 6811.71 | 52.09 | 5.15 | 0.25 | 18.98 | EB | After |
