# Supplementary material for: Trace element concentrations in forage seagrass species of Chelonia mydas along the Great Barrier Reef
Source: PLoS One. 2022 Jun 15;17(6):e0269806. doi: 10.1371/journal.pone.0269806 (PMC9200345; doi:10.1371/journal.pone.0269806)
Supplement: Attachment — Submitted filename: Response to Reviewers.docx [file pone.0269806.s002.docx]

| Responding to: | Point raised | Action taken? | Response |
| --- | --- | --- | --- |
| Academic editor | Manuscript formatting check | YES | I checked all formatting of the manuscript. I did not find guidelines regarding file naming in either of the formatting PDFs or the PLOS one submission guidelines. |
| Academic editor | Ethics statement must be in Methods section | YES | I moved the ethics statement to the methods section |
| Academic editor | Fig 1 map image citation | YES | Actions outlined in Esri ArcGIS permissions for use were adhered too. Acknowledgement of use of Arc GIS and base map citation included in figure caption. Specific written permission is not necessary to use a map created in ArcGIS software. |
| Academic editor | Captions for supporting information | YES | I added the title and caption to the supporting information, added the supporting information section at the end of the manuscript and cited the supporting information in the text as requested. |
| Academic editor | Review references | YES | All references were reviewed and are all cited within the text. No removal of references was conducted. |
| Reviewer #2 | Abstract is written well but it needs to make it concise for a gist. The sentence Toxic metal exposure is a threat to green sea turtles (Chelonia mydas) inhabiting and foraging in coastal seagrass meadows and is of particular concern in local bays of the Great Barrier Reef (GBR), as numerous sources of metal contaminants are located within the region. Seems to Introductory sentence. | YES | The highlighted sentence was moved and the abstract was altered to provide more of a concise read. |
| Reviewer #2 | Due to this, the primary source of metal element exposure for green turtles (Chelonia mydas) is through their diet. make it clear | YES | The statement in question was altered for clarification |
| Reviewer #2 | In addition to providing forage for C. mydas, seagrass fulfil several integral ecological 61 functions, such as sediment stabilization nutrient cycling the sequestration of 62 carbon Put the latest and relevant reference. | YES | 2021, 2022 or other newer relevant references were added where available and required. |
| Reviewer #2 | Gastric lavage sampling on a subsection of each population elaborate it? | YES | The statement was altered to provide clarification |
| Reviewer #2 | Once identified, seagrass samples were analysed for a suite of 10 metal elements using Inductively coupled Plasma Optical Emission Spectrometry to describe concentrations detected within coastal meadows along the Central GBR. Put the Modal and reference of analysis protocol | NO | No amendment was made in the section of note as the model and analysis protocol was later referenced in a more appropriate methods section. |
| Reviewer #2 | Fig 1 images are having poor visualization need to be update. Other figures are also poor in quality. | NO | The image quality of all figures is within the 300-600 dpi guideline for the journal and no comment on this was made by the academic editor or reviewer #1 |
| Reviewer #2 | The finding should be supported with latest reference there is no reference from 2021 and 2022 | YES | 2021, 2022 or other newer relevant references were added where available and required |
| Reviewer #2 | Conclusion Section needs to be summaries well. | YES | The conclusion has been added to and re-written to better convey the findings of the study and to summarise it best. |
| Reviewer #2 | Please double-check the manuscript for abbreviations. Abbreviations must be spelled out the first time they are mentioned in the abstract and starting again with the introduction section. | YES | The manuscript was checked for all abbreviations. All uses of abbreviations were correctly used following the first instance of being mentioned. |
| Reviewer #2 | Double-check that all references are cited within the text, and that all citations within the text have a corresponding reference. Double-check the spelling of the author names. Please ensure the abbreviation of the journal name. | YES | All references and citations were checked against the reference list and all are correct. Journal names were abbreviated for those references not already done so |

All changes are highlighted in yellow in the file named ‘Revised Manuscript with Track Changes’.

Reviewer #1 did not provide any comments to address and thus was not included in this table.
